# Effects of Lapatinib on HER2-Positive and HER2-Negative Canine Mammary Carcinoma Cells Cultured In Vitro

**DOI:** 10.3390/pharmaceutics13060897

**Published:** 2021-06-17

**Authors:** Antonio Fernando Leis-Filho, Patrícia de Faria Lainetti, Priscila Emiko Kobayashi, Carlos Eduardo Fonseca-Alves, Renée Laufer-Amorim

**Affiliations:** 1 Department of Veterinary Clinic, Sao Paulo State University-UNESP, Botucatu 18618-681, Brazil; a.leis@unesp.br (A.F.L.-F.); priscila.e.kobayashi@unesp.br (P.E.K.); 2 Department of Veterinary Surgery and Animal Reproduction, São Paulo State University-UNESP, Botucatu 18618-681, Brazil; patricia.lainetti@unesp.br (P.d.F.L.); carlos.e.alves@unesp.br (C.E.F.-A.); 3 Institute of Health Sciences, Paulista University-UNIP, Bauru 17048-290, Brazil

**Keywords:** dog, cancer, comparative oncology, molecular targets

## Abstract

HER2 is a prognostic and predictive marker widely used in breast cancer. Lapatinib is a tyrosine kinase inhibitor that works by blocking the phosphorylation of the receptor HER2. Its use is related to relatively good results in the treatment of women with HER2+ breast cancer. Thus, this study aimed to verify the effects of lapatinib on four canine primary mammary gland carcinoma cell cultures and two paired metastatic cell cultures. Cultures were treated with lapatinib at concentrations of 100, 500, 1000 and 3000 nM for 24 h and the 50% inhibitory concentration (IC_50_) for each cell culture was determined. In addition, a transwell assay was performed to assess the ability of lapatinib to inhibit cell migration. Furthermore, we verified *HER2* expression by RT-qPCR analysis of cell cultures and formalin-fixed paraffin-embedded tissues from samples corresponding to those used in cell culture. Lapatinib was able to inhibit cell proliferation in all cell cultures, but it was not able to inhibit migration in all cell cultures. The higher the expression of *HER2* in a culture, the more sensitive the culture was to treatment. This relationship may be an indication that the expression of *HER2* may be a predictive factor and opens a new perspective for the treatment of primary and metastatic mammary gland cancer.

## 1. Introduction

Mammary gland neoplasms exhibit several similarities between women and dogs, such as their high incidence, spontaneous appearance, common environmental risk factors, hormone receptor expression and neoplastic growth markers. Therefore, research related to one species may have aspects that can be studied in a comparative manner [1,2].

Estrogen receptor (ER), progesterone receptor (PR) and epidermal growth factor 2 (HER2) are among the most commonly used markers in human medicine with validated predictive value. Breast cancer in women can be classified according to the expression of the receptors ER, PR and HER2. Breast tumors can be divided into the molecular subtypes luminal A, luminal B, HER2 overexpressing and triple negative. Luminal A tumors are ER+, PR+ and HER2−, while luminal B tumors are divided into two groups and can be ER+, PR+ or − and HER2+ or ER+, PR+ or − and HER2−; HER2 tumors are ER−, PR− and HER2+, and triple-negative tumors are ER−, PR− and HER2− [3,4,5,6].

HER2 is a tyrosine kinase receptor member of the epidermal growth factor receptor (EGFR) family, which is composed of EGFR (also known as HER1), HER2, HER3 and HER4 [7]. The receptors in this family are located in the cell membrane, and when they bind to an external ligand, with the exception of HER2, which does not contain this binding site, they form dimers with other members of the family so that the internal phosphate domain is phosphorylated, triggering an intracellular response [8]. In addition, there is evidence that HER2 is the receptor that most often forms dimers with other members of the EGFR family [9]. Activation of these receptors is associated with increased survival, proliferation, and cell cycle progression [10].

HER2+ tumors have a prevalence of 13–15% among molecular subtypes, have a high Ki67 index and are generally high-grade tumors with a histology indicative of invasive carcinoma of no special type with aggressive disease characteristics; however, it is possible to use targeted therapies as treatment [5]. HER2 overexpression is related to shorter disease-free survival; reduced survival time; decreased ER and PR expression; increased cell proliferation, migration, tumor invasiveness, frequency of metastases, and angiogenesis; and decreased apoptosis [6]. Without targeted therapy, HER2+ cancers have a recurrence rate of up to 15% [4]. The survival rate of women diagnosed with tumors that overexpress HER2 is 92% over 20 months, 88% over 30 months, and decreases to almost 80% over 50 months [11]. In addition, brain metastasis of HER2+ breast tumors is common, affecting 50% of patients with metastatic disease [12].

Treatment of HER2-overexpressing tumors with targeted therapy produces a positive response when compared to treatments without the addition of targeted therapy [13]. Trastuzumab has been widely used, adding approximately 1 year to the disease-free interval of patients with this disease subtype [12], but even with this positive effect, some patients will still develop metastases or tumor recurrence after completion of the treatment [14,15]; therefore, new anti-HER2 drugs are being studied.

Lapatinib is an inhibitor of HER2 tyrosine kinase activity; it is considered a therapeutic alternative in the treatment of HER2+ neoplasms and acts as a reversible blocker of receptor phosphorylation. Lapatinib use is related to reduced disease progression and decreased axillary lymph node metastasis rates in women. Furthermore, because lapatinib is a small molecule, it can permeate the blood-brain barrier, making it an alternative therapy in cases of brain metastasis [16,17]. Lapatinib is an inhibitor of HER1/HER2 heterodimerization, and its action is independent of the HER1 status of tumors; it is also effective against HER2+ tumors resistant to treatment with trastuzumab [18,19] and is still an option in countries without access to other HER2 inhibitors and in cases of cardiac toxicity [19]. It should be used in combination, showing good results with capecitabine, and can be used as a second-line treatment for recurrent or metastatic HER2+ tumors [12,15,20]. In cell cultures of feline mammary cancer, lapatinib showed promising results, being able to inhibit cell viability when used as a single therapy and increase the action of anti-HER2 monoclonal antibodies such as trastuzumab and pertuzumab when used in combination. These results were observed both in cell cultures with high and low *HER2* expression [21,22].

According to Nguyen et al. [23], the overall survival rate of dogs with mammary gland tumors is 41.5% at one year and 54.1% at two years after surgery. The average survival times of animals after surgery for grade II and III tumors are 32.68 and 7.78 months, respectively [24]. In addition, the rate of metastasis of mammary gland tumors in dogs in general is 53% [25], and the recurrence rate of grade III tumors is 71.42% [24]. HER2+ mammary gland neoplasms in dogs represent approximately 8% of all the diagnosed molecular subtypes and are generally associated with a worse prognosis; larger tumors; higher histological grades, invasion, and proliferative indexes; and the presence of necrosis [25].

Canine mammary gland tumors are treated by surgical removal, and in some cases, the use of adjuvant chemotherapy is indicated, such as tumors with a more aggressive histological type, which have a higher rate of metastasis development. According to Cassali et al. [26], dogs that present with micropapillary carcinoma, solid carcinoma, carcinosarcoma, or pleomorphic lobular carcinoma, regardless of the degree, clinical stage and molecular subtype, are always indicated for chemotherapy. In addition, patients with tumors of a less aggressive subtype should undergo chemotherapy if the disease is grade III or above, and in any case when metastasis is present [26].

In veterinary medicine, there are no indications for selection of different chemotherapy protocols for different types of tumors, so carboplatin is widely applied in cases of female mammary tumors, regardless of the stage, histopathological type, molecular classification of the tumor and metastasis status [26]. The use of adjuvant carboplatin, when compared to surgical treatment alone, increased the survival time of animals with breast cancer [27], and the administration of carboplatin with mitoxantrone seems to promote a longer survival period, even if a study was carried out with a limited number of animals [28]. Clinical studies to evaluate the effectiveness of chemotherapy in animals are scarce, and therefore, information varies considerably.

To the best of our knowledge, the effect of lapatinib on canine mammary gland tumors has not been published. Since canine mammary gland tumors are very common, with most tumors being potentially malignant, and the standard therapy is radical mastectomy with no effective chemotherapy protocols for the treatment of animals with metastatic disease [26], this study aimed to evaluate the antitumor effect of lapatinib on HER2+ and HER2− primary and metastatic canine mammary gland carcinoma cells cultured in vitro.

## 2. Materials and Methods

### 2.1. Reagents

All reagents used were of high purity and purchased from GE Healthcare (Uppsala, Sweden), Sigma-Aldrich (São Paulo, Brazil), and Merck SA (São Paulo, Brazil); otherwise, the manufacturer is indicated. In addition, mammary epithelial cell growth medium (MEGM™; Lonza Inc., Allendale, NJ, USA), Dulbecco’s modified Eagle’s medium (DMEM; Lonza Inc.), fetal bovine serum (FBS; LGC Biotechnology, Cotia, SP, Brazil), Dulbecco’s phosphate-buffered saline (DPBS; Sigma Aldrich, St. Louis, MO, USA) and antibiotic/antimycotic solution (Thermo Fisher Scientific, Waltham, MA, USA) were used.

### 2.2. Inclusion Criteria

Samples that met all of the following criteria were included in this research: cell cultures of primary tumors that had paired formalin-fixed paraffin-embedded (FFPE) material and were classified according to Goldschmidt et al. [29], cell cultures characterized by cell phenotype and tumorigenicity in vitro, cell cultures with sufficient aliquots for triplicate analysis in all experiments and free of bacterial, mycoplasma and/or fungal contamination. Cell lines were previously established and characterized [30]. A total of six cell lines met the criteria. Among the cell lines, four cell lines were primary mammary carcinoma cells (UNESP-CM1, UNESP-CM5, UNESP-CM9 and UNESP-CM60) and two were mammary carcinoma metastases (UNESP-MM1 and UNESP-MM4) (Supplementary Materials Table S1)

### 2.3. In Silico Analysis of HER2 Homology

To assess the three-dimensional protein structure homology between human and canine HER2, an in silico analysis was performed. The amino acid sequences of the human (UniProtKB/Swiss-Prot: P04626.1) and canine (NCBI Reference Sequence: NP_001003217.2) proteins were obtained from NCBI GenBank (https://www.ncbi.nlm.nih.gov/genbank/ (accessed on 14 May 2021)). Prediction analysis of the homology of the three-dimensional structure of the HER2 protein was performed using Swiss software (Swiss Institute of Bioinformatics, Basel, Switzerland) (https://swissmodel.expasy.org/ (accessed on 14 May 2021)).

### 2.4. Experimental Groups

The determination of the sample number in the study was based on that described by Lazic et al. [31]. Four samples of primary mammary carcinoma and two samples of metastatic tumors were selected and divided into two groups with three samples each according to the HER2 expression in the tissue: one group contained the HER2+ samples (UNESP-CM1, UNESP-CM9 and UNESP-CM60), and the other contained the HER2- samples (UNESP-MM1, UNESP-MM4 and UNESP-CM5). Both groups (HER2+ and HER2− cells) were treated with lapatinib. The cell culture UNESP-CM1 was used on the 15th passage, UNESP-CM5 on the 16th, UNESP-CM9 on the 17th, UNESP-CM60 on the 35th, UNESP-MM1 on the 19th, and UNESP-MM4 on the 22nd. The passages used were chosen according to cell stability and aliquot availability [30].

### 2.5. HER2 Immunoreactivity

FFPE samples, paired with the cell cultures, were cut on a microtome (5 μm), transferred to positively charged slides (StarFrost™, Braunschweig, Germany) and dewaxed. The slides were subjected to antigen retrieval with citrate buffer (pH 6.0) in a pressure cooker (Pascal, Dako, Agilent Technologies, Santa Clara, CA, USA). Endogenous peroxidase activity was blocked with 8% hydrogen peroxide in methanol for 20 min and blocking of nonspecific proteins was carried out with 8% skim milk for 60 min; both steps were performed at room temperature. Antibody detection was performed using a polymer system (EnVision™ Dako, Agilent Technologies, Santa Clara, CA, USA). The anti-HER2 antibody from the Herceptest^TM^ Kit (Dako, Carpinteria, CA, USA) was used according to the manufacturer’s instructions, and 3 3’-diaminobenzidine (EnVision™ FLEX, High pH, Dako, Agilent Technologies, Santa Clara, CA, USA) was used as the chromogen. Counterstaining was performed with Harris hematoxylin. Negative controls were generated by omission of the primary antibody. The positive control used was provided with the manufacturer’s kit. To consider canine samples HER2+, the criteria described in the commercial kit approved by the US Food and Drug Administration, Herceptest^TM^, were followed. In summary, a score of 0 was given when no staining was observed or membrane staining was observed in <10% of tumor cells; a score of 1+ was given when faint or barely perceptible membrane staining was detected in >10% of tumor cells and the cells exhibited incomplete membrane staining; a score of 2+ was given when weak to moderate complete membrane staining was observed in >10% of tumor cells; and a score of 3+ was given when strong complete membrane staining was observed in >10% of tumor cells.

**Figure 1:**
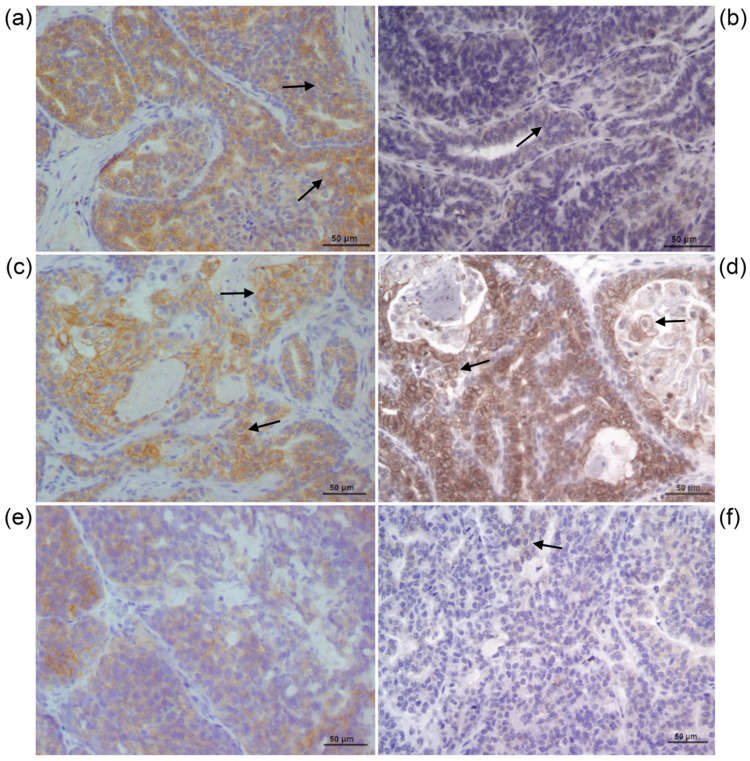
Immunohistochemical analysis of HER2 in samples of canine mammary carcinoma. Cell membrane staining indicates a positive cell for HER2 expression (arrows). (**a**) UNESP-CM1; (**b**) UNESP-CM5; (**c**) UNESP-CM9; (**d**) UNESP-CM60; (**e**) UNESP-MM1; (**f**) UNESP-MM4.

Six samples were submitted for immunohistochemistry. Among these, three were classified as negative for HER2 (two with a score of 1+ and one with a score of 0), and three were considered HER2 positive (three with a score of 3+) (Table 1, Figure 1).

### 2.6. Lapatinib Treatment and Evaluation of Cellular Metabolic Activity

To evaluate the antitumor effect of lapatinib (50% inhibitory concentration, IC_50_), an in vitro MTT assay was performed. The drug was diluted in DMSO to a concentration of 2 mg/mL, and the highest dilution was used as the parameter for the control containing DMSO (at the same concentration as the highest concentration of lapatinib). Cells were cultured according to Lainetti et al. [30].

The concentrations of lapatinib tested were 100, 500, 1000, and 3000 nM [32], and cells were treated for 24 h to determine the IC_50_. Tumor cells were seeded in 96-well plates at a concentration of 10,000 cells/well and incubated for 24 h at 37 °C in DMEM containing 5% FBS. Subsequently, lapatinib was added to fresh medium without FBS, and the culture was incubated for an additional 24 h in a humidified atmosphere with 5% CO_2_.

After 24 h of cultivation, 10 μL of MTT solution (Sigma Aldrich, St. Louis, MO, USA) at a concentration of 0.5 mg/mL diluted in DPBS was added to each well, and the plate was incubated at 37 °C for 4 h. After the incubation, the formazan resulting from MTT cleavage was solubilized with DMSO. After 10 min of homogenization, the absorbance at 550 nm was determined with a microplate reader (Biochrom Asys Expert Plus Microplate Reader, Biochrom Ltd., Harvard Bioscience, Holliston, MA, USA). Based on the test results, the IC_50_ [33] was determined using the formula: % of antioxidant activity = 100 − (absorbance of the treated sample − absorbance of the blank) × 100/absorbance of the control cells, where the blank was DPBS and the cells in the control group were not treated with lapatinib.

### 2.7. RNA Extraction from Paraffin-Embedded Tissue and Cell Culture Samples and RT-qPCR Analysis

mRNA was extracted from FFPE samples and the corresponding cell cultures for *HER2* gene expression analysis. For mRNA extraction from the paraffin-embedded tissues, samples were cut on a microtome, and three sections of 10 micrometers were macrodissected and placed in 1.5 mL tubes. The extraction protocol of the RecoverAll™ Total Nucleic Acid Isolation Kit for FFPE (Invitrogen, Carlsbad, CA, USA) was followed. At the end of the protocol, the samples were treated with DNase to purify the RNA, and the RNA concentration and purity were measured by determining the A280 absorbance and A260/A280 ratio on a spectrophotometer (NanoDropTM, ND-8000, Thermo Scientific, Waltham, MA, USA).

mRNA extraction from the six cell cultures was carried out in triplicate, and for this purpose, cryopreserved cells were thawed in a water bath at 37 °C, centrifuged (450 g, 5 min) and resuspended in DMEM supplemented with 1% antibiotic/antimycotic solution and 10% FBS. The cells were transferred to 6-well plates and cultured until they reached a minimum confluence of 70%. Then, they were washed 3 times with DPBS in an ice bath.

mRNA extraction followed the protocol recommended by the manufacturer (RNeasy Mini Kit, Qiagen, Hilden, Germany). The concentration and purity of the extracted mRNA were evaluated by determining the A280 absorbance and A260/A280 ratio on a spectrophotometer (NanoDropTM, ND-8000, Thermo Scientific, Waltham, MA, USA). To eliminate any contamination with genomic DNA, the total extracted RNA was treated with 1 U of DNase I amplification grade (Life Technologies, Carlsbad, CA, USA) in 10× DNase I reaction buffer and 25 mM EDTA, pH 8.0.

For both mRNA extracted from paraffin embedded tissue and that extracted from cells in culture, RNA was reverse transcribed into cDNA with 1 μg of total RNA and the Super-script III™ Reverse Transcriptase enzyme (Invitrogen). For this protocol, 1 μL of OligodT (500 μg/mL), 1 μL of random primers (100 μg/mL), 1 μL of dNTPs and RNase-free water were used. The mixture was heated to 65 °C for 5 min, 4 μL of 5× First-Strand Buffer transcription buffer (250 mM Tris-HCl, pH 8.3; 375 mM KCl; and 15 mM MgCl_2_) and 1 μL of 0.1 M DTT were added, and then 1 μL of the Super-script III enzyme (200 U/μL) was added, with the final volume of the mixture being 19 μL. The mixtures were incubated at 25 °C for 5 min and then at 50 °C for 1.5 h, followed by incubation at 70 °C for 15 min. Reactions were carried out on a PTC-100 thermocycler (Peltier-EffectCycling-MJ Research). At the end of transcription, cDNA was stored at −20 °C.

RT-qPCR amplifications were evaluated on an automatic thermocycler (QuantStudio™ 12K Flex Real-Time PCR System, 4471087, Applied Biosystems™, ThermoFisher Scientific, Carlsbad, CA, USA) and processed by the detection system after a variable number of cycles in the exponential phase.

The values obtained for expression in all samples were normalized as the ratio between the gene of interest (*HER2*) and reference genes *hypoxanthine phosphoribosyltransferase 1 (HPRT)*, *ribosomal protein S5* (*RPS5*) and *ribosomal protein S19* (*RPS19*) (selected in previous studies by the group). The primers used in this reaction are listed in Table 2.

Transcript expression was analyzed as the relative quantification (RQ) of the RNA expression in a sample using the formula 2^−ΔΔCt^ [34]. The RQ of each sample was determined by comparison with normal FFPE mammary tissue samples.

### 2.8. Cell Migration Assay

For evaluation of the effect of lapatinib on cell migration, a transwell assay was used. All cell cultures evaluated in this assay went through a 24 h period “starvation” period in medium containing 0.2% FBS. After they were treated with trypsin and incubated in a humidified atmosphere containing 5% CO_2_ at 37 °C for 5 min, the cells were loosened from the bottom of the bottle. Then, the trypsin was inactivated with MEGM™ supplemented with 5% FBS. Samples were centrifuged (450 g, 5 min) to remove the medium with a high concentration of FBS and then resuspended in MEGM™ supplemented with 0.2% FBS. Two hundred microliters of each cell culture suspension was placed in inserts (Greiner Bio-One, Kremsmünster, Austria) with a porous membrane of 8 µM at a concentration of 1 × 10^6^ cells/mL, and each insert was placed in a well of a 24-well plate containing MEGM™ supplemented with 10% FBS.

Each cell culture was placed in six different inserts, three were used as control wells and three were used as treatment wells. For treatment wells, lapatinib was added to the insert at the IC_50_ of each culture. After 24 h, the inserts were removed from the plate and placed in a new 24-well plate containing preheated trypsin. The samples were incubated in trypsin for 10 min in a humidified atmosphere containing 5% CO_2_ at 37 °C. After that period, the trypsin-treated cells that released from the bottom of the inserts were placed in a Neubauer chamber, and the cells were counted.

### 2.9. Statistical Analysis

RQs were evaluated to determine the correlation of HER2 expression in paraffin-embedded tissue and that in culture samples by Spearman’s correlation analysis. In addition, the Mann–Whitney test was used to assess the difference in HER2 expression between paraffin-embedded tissue and cell culture samples. Data obtained from the migration assay were statistically analyzed using the Mann–Whitney test. MTT results were analyzed using the Mann–Whitney test. The results of MTT and RT-qPCR assay were evaluated for correlations using the Spearman test. Statistical differences were considered significant when *p* < 0.05.

## 3. Results

### 3.1. HER2 Homology

**Figure 2:**
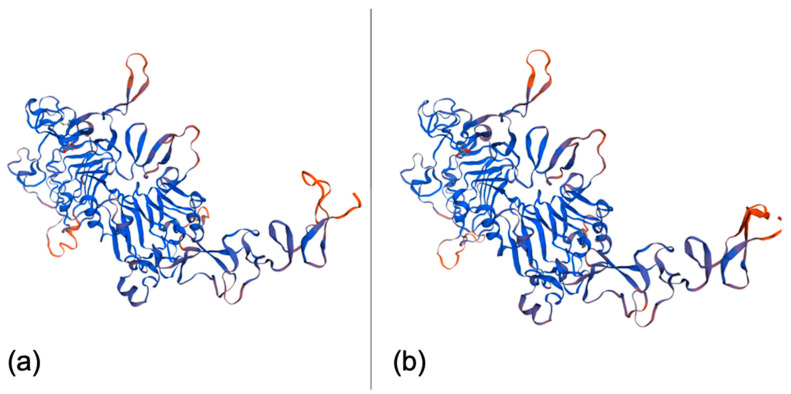
(**a**) 3D model of human HER2 generated from the UniProtKB/Swiss-Prot sequence P04626.1. (**b**) Canine HER2 3D model generated from the NCBI reference sequence NP_001003217.2. Both images were generated using the online tool Swiss model (Swiss Institute of Bioinformatics, Basel, Switzerland) (https://swissmodel.expasy.org/ (accessed on 14 May 2021)).

When compared on the Swiss model platform (Swiss Institute of Bioinformatics, Basel, Switzerland) (https://swissmodel.expasy.org/ (accessed on 14 May 2021)), the three-dimensional structures of canine and human HER2 showed 95% homology (Figure 2), demonstrating high homology between the human and canine HER2 proteins.

### 3.2. Gene Expression

There was no statistical correlation between the expression of *HER2* in paraffin-embedded samples and the immunohistochemical score (*p* = 0.13).

### 3.3. Cell Viability

**Figure 3:**
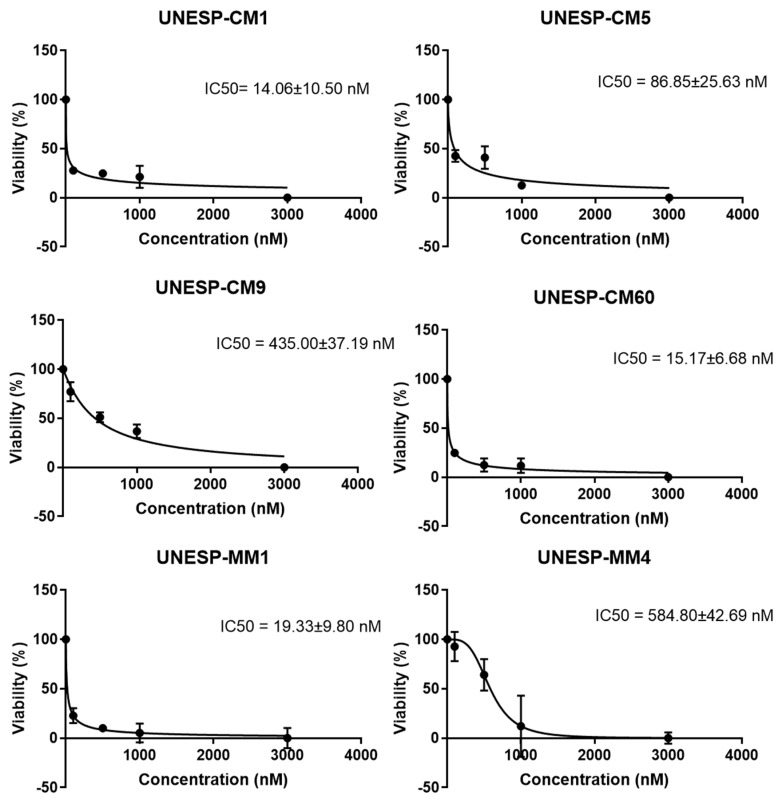
Percentage of viable cells in an MTT assay of primary cultures of canine breast carcinoma (UNESP-CM1, UNESP-CM5, UNESP-CM9 UNESP-CM60) and metastases (UNESP-MM1 and UNESP-MM4) treated with lapatinib at 100, 500, 1000 or 3000 nM for 24 h.

All primary cultures showed decreased cellular metabolic activity at all tested drug concentrations in an MTT assay. The IC_50_ values of the six cell cultures were between 14.06 nM and 584.80 nM (Figure 3).

**Figure 4:**
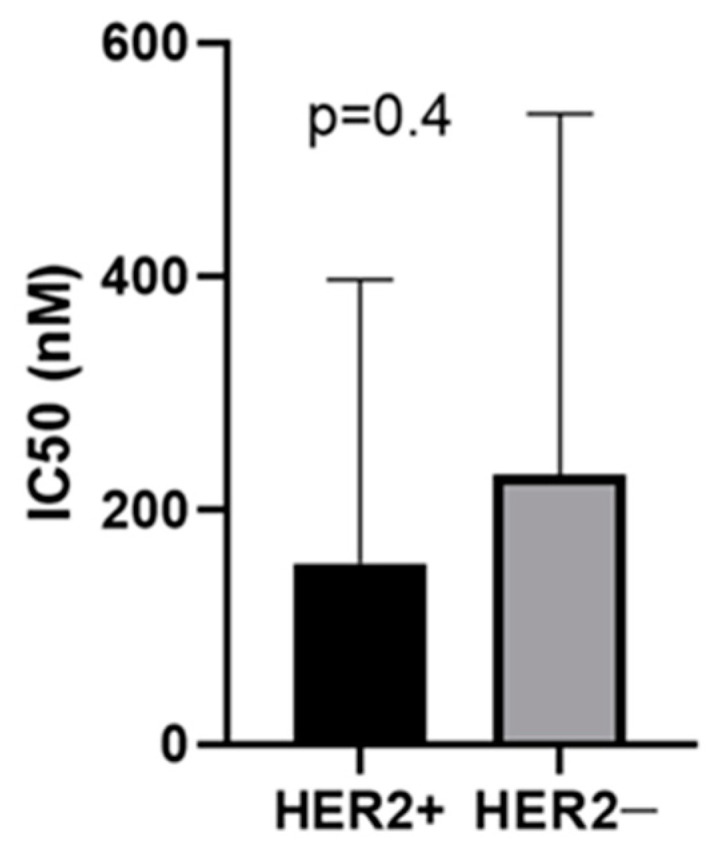
Comparison by Mann–Whitney test of the IC_50_ values of the HER2+ and HER2− groups (*p* = 0.4).

There was no significant difference between the IC_50_ of the HER2+ group and that of the HER2− group (*p* = 4) (Figure 4), and there was no significant difference in the IC_50_ among the primary and metastatic cell lines (*p* = 0.53).

### 3.4. Correlation between HER2 Expression and the IC_50_

Spearman’s correlation analysis revealed a negative correlation (*p* = 0.04) between the RQ of cell culture samples and the IC_50_; that is, the higher the HER2 expression of a sample was, the lower the IC_50_ of lapatinib for that culture.

### 3.5. Cell Migration

**Figure 5:**
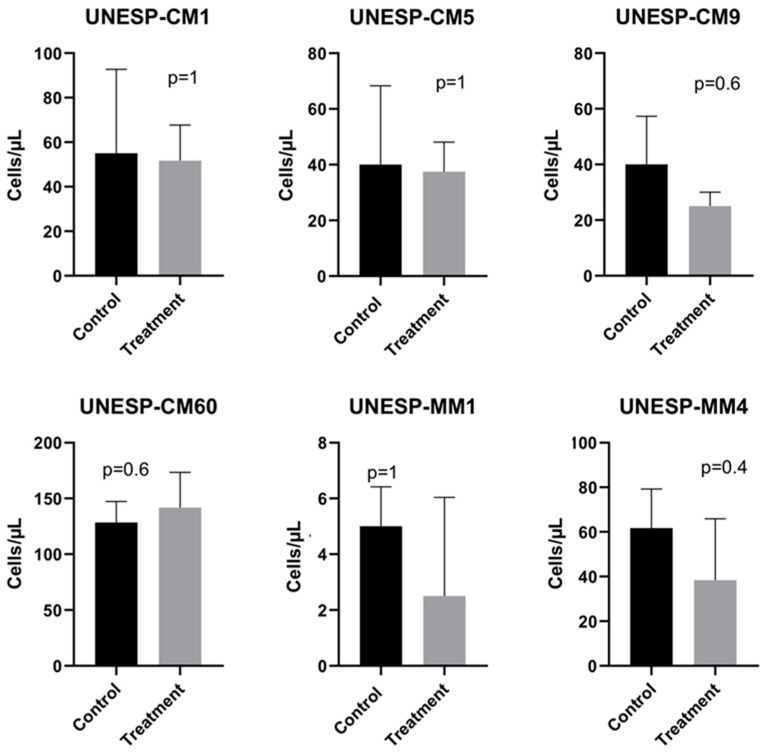
Concentration (cells/µL) of cells in the wells of each cell culture after the migration assay protocol. The Mann–Whitney test was used to compare the migration of the control and treatment groups.

In a migration assay, there was no significant difference between the group treated with lapatinib and the control group in any cell culture (Figure 5). Additionally, there was no difference when grouping the cells by HER2 expression and their origin (non-metastatic primaries, metastatic primaries and metastases) as seen on the Supplementary Materials Figures S1 and S2.

## 4. Discussion

In clinical practice, trastuzumab is considered the first-line choice for treatment of human breast cancer overexpressing HER2 [35]. However, advanced tumors can become resistant to trastuzumab, and in these cases, the use of lapatinib alone or in combination leads to a better antitumor response [36,37,38]. Female dogs can be considered a model of human breast cancer, but only for some specific subtypes, such as the micropapillary, solid, and anaplastic subtypes [39] and inflammatory breast cancer [40].

Singer et al. [41] studied the homology between canine and human HER2 and predicted the binding of anti-HER2 drugs with canine HER2. The HER2 proteins of humans and dogs showed high homology of 92.31% in the amino acid sequence. In addition, important sequences, such as the binding region for trastuzumab and cetuximab, were conserved in canine HER2. Moreover, there was predicted binding between these monoclonal antibodies and canine HER2.

In our assessment of the three-dimensional homology of the structures of human and canine HER2, the homology was even greater, with 95% similarity, so it is very likely that polyclonal antibodies developed to recognize human HER2 can recognize canine HER2. Thus, this similarity in proteins is also important, as it indicates that targeted therapies developed for human use should be applicable in dogs.

Lapatinib efficiently inhibited the proliferation of HER2+ and HER2− canine mammary carcinoma cell cultures in vitro. Interestingly, the cell lines exhibited dose-dependent cell viability. Thus, higher concentrations produced lower cell viability. This result agrees with the findings in humans, which showed that lapatinib was able to reduce the viability and proliferation of breast cancer cells and induce breast cancer cell death [41,42,43,44]. Similar results were also reported in feline mammary cancer cell cultures, where lapatinib both alone and in association with other drugs such as rapamycin, trastuzumab and pertuzumab, was able to inhibit cell proliferation [21,22].

In assessing the ability of lapatinib to inhibit the migration of canine mammary cancer cells, we confirmed that just as in human breast cancer cells, lapatinib alone is not able to satisfactorily inhibit migration regardless of the cell sensitivity to the drug [45]. However, when associated with isothiocyanates, compounds present in abundance in cruciferous vegetables, lapatinib was shown to inhibit the migration of breast cancer cells in women [45,46]. Lapatinib in combination with foretinib, an inhibitor of hepatocyte growth factor receptor, was also found to inhibit migration in triple-negative human breast cancer cell lines due to its action on EGFR [47]. These findings create interesting possibilities for future studies in canine mammary gland tumors assessing the combination of other pharmaceutical compounds with lapatinib.

In our study, lapatinib inhibited cell viability in samples of primary and metastatic neoplasms even with low or negative HER2 expression. This action is also observed in humans and makes lapatinib an excellent therapeutic option in the treatment of metastatic breast neoplasms [48,49]. Therefore, lapatinib is a possible alternative that should be studied for the treatment of dogs with metastatic disease. Another important factor to be considered in the use of lapatinib to treat metastases is its good ability to penetrate different body tissues due to the small size of the molecule [8].

Although our study focused on HER2+ neoplasms, lapatinib has also been studied as a possible therapeutic alternative for triple-negative breast neoplasms, which generally show increased EGFR expression [47,48,50]. Studies on this subtype have demonstrated the ability of lapatinib, when combined with other drugs, to inhibit the migration of triple-negative human breast cancer cells [47]. In addition, in a study of human patients with metastatic triple-negative breast cancer treated with lapatinib and veliparib, 35% (6/17) of the patients responded to the therapy, with less than 10% of the patients having adverse effects, all of which were moderate [48].

Therefore, although lapatinib inhibited cell proliferation in all cell cultures, even in three of them considered negative for HER2, we emphasize that five of the six tumors had HER2 expression, even if at a low level. Thus, the drug may have acted on the few HER2 molecules present, and in the case of the cell line UNESP-MM1, the action could be related to binding to EGFR [16,51], this ability of lapatinib to inhibit EGFR phosphorylation has already been described in feline mammary cancer cells [22]. In addition, similar to the activity of any tyrosine kinase inhibitor, nonspecific binding with other tyrosine kinase receptors can occur [16,51].

The negative correlation between the expression of HER2 in cells and the IC_50_, that is, the higher the expression of HER2 in a cell culture was, the lower the IC_50_ of lapatinib of that sample, is an indication that, as in humans, lapatinib acts mainly on HER2 in canine cells, reducing the viability of neoplastic cells [32,52]. In addition, the correlation between HER2 expression in cultured cells and cell sensitivity to lapatinib is a strong indication that the level of expression of this receptor may be a predictive factor for therapies using lapatinib, which opens the door for further in vivo studies in dogs.

## 5. Conclusions

Lapatinib was able to reduce the viability of primary and metastatic canine mammary carcinoma cells cultured in vitro, and its effectiveness was directly linked to the expression of HER2, which opens a perspective for the treatment of animals with both primary mammary neoplasms and metastasis, especially those that overexpress HER2.

## Figures and Tables

**Table 1:** Classification of samples used in the study according to the expression of HER2 determined by immunohistochemistry using Herceptest^TM^. The samples received scores according to the recommendations of the kit used.

Cell Type	UNESP-MM1	UNESP-MM4	UNESP-CM1	UNESP-CM5	UNESP-CM9	UNESP-CM60
HER2 expression	0	1+	3+	1+	3+	3+

**Table 2:** Oligonucleotide sequences of the primers used for RT-qPCR.

Access Gene Symbol ^1^	Oligonucleotide Sequence (5′ > 3′)
*HPRT*	Forward primer (5′-3′) AGCTTGCTGGTGAAAAGGAC
	Reverse primer (3′-5′) TTATAGTCAAGGGCATATCC
*RPS19*	Forward primer (5′-3′) CCTTCCTCAAAAAGTCTGGG
	Reverse primer (3′-5′) GAACGAGGGATGCTACTCTTG
*RPS5*	Forward primer (5′-3′) TCACTGGTGAGAACCCCCT
	Reverse primer (3′-5′) TCACTGGTGAGAACCCCCT
*HER2*	Forward primer (5′-3′) GCTCTGGAGGGAGTCACAGGTTA
	Reverse primer (3′-5′) ACTGAGGTTAGGCAGGCTGTCT

^1^ GenBank (www.ncbi.nlm.nih.gov (accessed on 12 January 2021)).

## Supplementary Materials

The following are available online at https://www.mdpi.com/article/10.3390/pharmaceutics13060897/s1, Figure S1: Mann-Whitney analysis of cell migration. There was no statistical difference in any analysis (a): All cell cultures. (b): HER2+ cell cultures. (c): HER2− cell cultures, Figure S2: ANOVA evaluation of cell culture migration when grouped into non-metastatic primaries, metastatic primaries and metastases. Non-metastatic primaries versus metastatic primaries *p* = 0.2873; Non-metastatic primaries versus metastases *p* = 1; Metastatic primaries versus metastases *p* = 0.1822. Table S1. Clinical data from animals with mammary carcinoma used for primary cell culture.

## References

[1] Yu R.M.C., Cheah Y.K. (2017). The roles of miRNAs in human breast cancer and canine mammary tumor. Appl. Cancer Res..

[2] Sahabi K., Selvarajah G.T., Abdullah R., Cheah Y.K., Tan G.C. (2018). Comparative aspects of microRNA expression in canine and human cancers. J. Veter Sci..

[3] Sorlie T., Perou C.M., Tibshirani R., Aas T., Geisler S., Johnsen H., Hastie T., Eisen M.B., van de Rijn M., Jeffrey S.S. (2001). Gene expression patterns of breast carcinomas distinguish tumor subclasses with clinical implications. Proc. Natl. Acad. Sci. USA.

[4] Fragomeni S.M., Sciallis A., Jeruss J.S. (2018). Molecular Subtypes and Local-Regional Control of Breast Cancer. Surg. Oncol. Clin. N. Am..

[5] Harbeck N., Penault-Llorca F., Cortes J., Gnant M., Houssami N., Poortmans P., Ruddy K., Tsang J., Cardoso F. (2019). Breast cancer. Nat. Rev. Dis. Prim..

[6] Ross J.S., Slodkowska E.A., Symmans W.F., Pusztai L., Ravdin P.M., Hortobagyi G.N. (2009). The HER-2 Receptor and Breast Cancer: Ten Years of Targeted Anti–HER-2 Therapy and Personalized Medicine. Oncology.

[7] Anderson N.G., Ahmad T. (2002). ErbB receptor tyrosine kinase inhibitors as therapeutic agents. Front. Biosci..

[8] Roskoski R. (2004). The ErbB/HER receptor protein-tyrosine kinases and cancer. Biochem. Biophys. Res. Commun..

[9] Arkhipov A., Shan Y., Kim E.T., O Dror R., E Shaw D. (2013). Her2 activation mechanism reflects evolutionary preservation of asymmetric ectodomain dimers in the human EGFR family. eLife.

[10] Holbro T. (2003). The ErbB receptors and their role in cancer progression. Exp. Cell Res..

[11] Arciero C.A., Guo Y., Jiang R., Behera M., O’Regan R., Peng L., Li X. (2019). ER+/HER2+ Breast Cancer Has Different Metastatic Patterns and Better Survival Than ER−/HER2+ Breast Cancer. Clin. Breast Cancer.

[12] Waks A.G., Winer E.P. (2019). Breast Cancer Treatment: A Review. JAMA.

[13] Howlader N., Cronin K.A., Kurian A.W., Andridge R. (2018). Differences in Breast Cancer Survival by Molecular Subtypes in the United States. Cancer Epidemiol. Biomark. Prev..

[14] Escrivá-De-Romaní S., Arumí M., Bellet M., Saura C. (2018). HER2-positive breast cancer: Current and new therapeutic strategies. Breast.

[15] Kreutzfeldt J., Rozeboom B., Dey N., De P. (2020). The trastuzumab era: Current and upcoming targeted HER2+ breast cancer therapies. Am. J. Cancer Res..

[16] Arteaga C.L., Sliwkowski M.X., Osborne C.K., Perez E.A., Puglisi F., Gianni L. (2011). Treatment of HER2-positive breast cancer: Current status and future perspectives. Nat. Rev. Clin. Oncol..

[17] Patel T.A., Ensor J.E., Creamer S.L., Boone T., Rodriguez A.A., Niravath P.A., Darcourt J.G., Meisel J.L., Li X., Zhao J. (2019). A randomized, controlled phase II trial of neoadjuvant ado-trastuzumab emtansine, lapatinib, and nab-paclitaxel versus trastuzumab, pertuzumab, and paclitaxel in HER2-positive breast cancer (TEAL study). Breast Cancer Res..

[18] Vernieri C., Milano M., Brambilla M., Mennitto A., Maggi C., Cona M.S., Prisciandaro M., Fabbroni C., Celio L., Mariani G. (2020). The trastuzumab era: Current and upcoming targeted HER2+ breast cancer therapies. Am. J. Cancer Res..

[19] Bredin P., Walshe J.M., Denduluri N. (2020). Systemic therapy for metastatic HER2-positive breast cancer. Semin. Oncol..

[20] Kunte S., Abraham J., Montero A.J. (2020). Novel HER2–targeted therapies for HER2–positive metastatic breast cancer. Cancer.

[21] Gameiro A., Nascimento C., Correia J., Ferreira F. (2021). HER2-Targeted Immunotherapy and Combined Protocols Showed Promising Antiproliferative Effects in Feline Mammary Carcinoma Cell-Based Models. Cancers.

[22] Gameiro A., Almeida F., Nascimento C., Correia J., Ferreira F. (2021). Tyrosine Kinase Inhibitors Are Promising Therapeutic Tools for Cats with HER2-Positive Mammary Carcinoma. Pharmaceutics.

[23] Nguyen F., Peña L., Ibisch C., Loussouarn D., Gama A., Rieder N., Belousov A., Campone M., Abadie J. (2018). Canine invasive mammary carcinomas as models of human breast cancer. Part 1: Natural history and prognostic factors. Breast Cancer Res. Treat..

[24] Peña L., De Andrés P.J., Clemente M., Cuesta P., Pérez-Alenza M.D. (2012). Prognostic Value of Histological Grading in Noninflammatory Canine Mammary Carcinomas in a Prospective Study with Two-Year Follow-Up. Veter Pathol..

[25] Gama A., Alves A., Schmitt F. (2008). Identification of molecular phenotypes in canine mammary carcinomas with clinical implications: Application of the human classification. Virchows Archiv..

[26] Cassali G., Damasceno K., Bertagnolli A., Estrela-Lima A., Lavalle G., Santis G., Nardi A., Fernandes C., Cogliati B., Sobral R. (2017). Consensus regarding the diagnosis, prognosis and treatment of canine mammary tumors: Benign mixed tumors, carcinomas in mixed tumors and carcinosarcomas. Braz. J. Veter Pathol..

[27] Lavalle G.E., Campos C.B., Bertagnolli A.C., Cassali G.D. (2012). Canine malignant mammary gland neoplasias with advanced clinical staging treated with carboplatin and cyclooxygenase inhibitors. In Vivo.

[28] Tran C.M., Moore A.S., Frimberger A. (2016). Surgical treatment of mammary carcinomas in dogs with or without postoperative chemotherapy. Veter Comp. Oncol..

[29] Goldschmidt M., Peña L., Rasotto R., Zappulli V. (2011). Classification and Grading of Canine Mammary Tumors. Veter Pathol..

[30] Lainetti P.D.F., Brandi A., Filho A.F.L., Prado M.C.M., Kobayashi P.E., Laufer-Amorim R., Fonseca-Alves C.E. (2020). Establishment and Characterization of Canine Mammary Gland Carcinoma Cell Lines with Vasculogenic Mimicry Ability in vitro and in vivo. Front. Veter Sci..

[31] Lazic S.E., Clarke-Williams C.J., Munafò M.R. (2018). What exactly is ‘N’ in cell culture and animal experiments?. PLoS Biol..

[32] Nahta R., Yuan L.X., Du Y., Esteva F.J. (2007). Lapatinib induces apoptosis in trastuzumab-resistant breast cancer cells: Effects on insulin-like growth factor I signaling. Mol. Cancer Ther..

[33] Negri J.M., McMillin D.W., Delmore J., Mitsiades N., Hayden P., Klippel S., Hideshima T., Chauhan D., Munshi N.C., Buser C.A. (2009). In vitroanti-myeloma activity of the Aurora kinase inhibitor VE-465. Br. J. Haematol..

[34] Livak K.J., Schmittgen T.D. (2001). Analysis of relative gene expression data using real-time quantitative PCR and the 2^–ΔΔCT^ Method. Methods.

[35] Maximiano S., Magalhães P., Guerreiro M.P., Morgado M. (2016). Trastuzumab in the Treatment of Breast Cancer. BioDrugs.

[36] Riera R., De Soárez P.C., Puga M.E.D.S., Ferraz M.B. (2009). Lapatinib for treatment of advanced or metastasized breast cancer: Systematic review. Sao Paulo Med. J..

[37] Opdam F.L., Guchelaar H.-J., Beijnen J.H., Schellens J.H. (2012). Lapatinib for Advanced or Metastatic Breast Cancer. Oncology.

[38] Yang F., Huang X., Sun C., Li J., Wang B., Yan M., Jin F., Wang H., Zhang J., Fu P. (2020). Lapatinib in combination with capecitabine versus continued use of trastuzumab in breast cancer patients with trastuzumab-resistance: A retrospective study of a Chinese population. BMC Cancer.

[39] Al-Mansour M.A., Kubba M.A., Al-Azreg S.A., Dribika S.A. (2018). Comparative histopathology and immunohistochemistry of human and canine mammary tumors. Open Veter J..

[40] De Andrés P.J., Illera J.C., Cáceres S., Díez L., Pérez-Alenza M.D., Peña L. (2013). Increased levels of interleukins 8 and 10 as findings of canine inflammatory mammary cancer. Veter Immunol. Immunopathol..

[41] Singer J., Weichselbaumer M., Stockner T., Mechtcheriakova D., Sobanov Y., Bajna E., Wrba F., Horvat R., Thalhammer J.G., Willmann M. (2012). Comparative oncology: ErbB-1 and ErbB-2 homologues in canine cancer are susceptible to cetuximab and trastuzumab targeting. Mol. Immunol..

[42] Guan M., Tong Y., Guan M., Liu X., Wang M., Niu R., Zhang F., Dong N., Shao J., Zhou Y. (2018). Lapatinib Inhibits Breast Cancer Cell Proliferation by Influencing PKM2 Expression. Technol. Cancer Res. Treat..

[43] Showalter L.E., Oechsle C., Ghimirey N., Steele C., Czerniecki B.J., Koski G.K. (2019). Th1 cytokines sensitize HER-expressing breast cancer cells to lapatinib. PLoS ONE.

[44] Ma S., Dielschneider R.F., Henson E.S., Xiao W., Choquette T.R., Blankstein A.R., Chen Y., Gibson S.B. (2017). Ferroptosis and autophagy induced cell death occur independently after siramesine and lapatinib treatment in breast cancer cells. PLoS ONE.

[45] Kaczyńska A., Herman-Antosiewicz A. (2016). Combination of lapatinib with isothiocyanates overcomes drug resistance and inhibits migration of HER2 positive breast cancer cells. Breast Cancer.

[46] Wu X., Zhou Q.-H., Xu K. (2009). Are isothiocyanates potential anti-cancer drugs?. Acta Pharmacol. Sin..

[47] Simiczyjew A., Dratkiewicz E., Van Troys M., Ampe C., Styczeń I., Nowak D. (2018). Combination of EGFR Inhibitor Lapatinib and MET Inhibitor Foretinib Inhibits Migration of Triple Negative Breast Cancer Cell Lines. Cancers.

[48] Stringer-Reasor E.M., May J.E., Olariu E., Caterinicchia V., Li Y., Chen D., Della Manna D.L., Rocque G.B., Vaklavas C., Falkson C.I. (2021). An open-label, pilot study of veliparib and lapatinib in patients with metastatic, triple-negative breast cancer. Breast Cancer Res..

[49] Ma F., Ouyang Q., Li W., Jiang Z., Tong Z., Liu Y., Li H., Yu S., Feng J., Wang S. (2019). Pyrotinib or Lapatinib Combined With Capecitabine in HER2–Positive Metastatic Breast Cancer with Prior Taxanes, Anthracyclines, and/or Trastuzumab: A Randomized, Phase II Study. J. Clin. Oncol..

[50] Abo-Zeid M.A., Abo-Elfadl M.T., Gamal-Eldeen A.M. (2019). Evaluation of lapatinib cytotoxicity and genotoxicity on MDA-MB-231 breast cancer cell line. Environ. Toxicol. Pharmacol..

[51] Broekman F. (2011). Tyrosine kinase inhibitors: Multi-targeted or single-targeted?. World J. Clin. Oncol..

[52] Konecny G.E., Pegram M.D., Venkatesan N., Finn R., Yang G., Rahmeh M., Untch M., Rusnak D.W., Spehar G., Mullin R.J. (2006). Activity of the Dual Kinase Inhibitor Lapatinib (GW572016) against HER-2-Overexpressing and Trastuzumab-Treated Breast Cancer Cells. Cancer Res..

