# Supplementary material for: Effects of Lapatinib on HER2-Positive and HER2-Negative Canine Mammary Carcinoma Cells Cultured In Vitro
Source: Pharmaceutics. 2021 Jun 17;13(6):897. doi: 10.3390/pharmaceutics13060897 (PMC8235449; doi:10.3390/pharmaceutics13060897)
Supplement: Supplementary file 1 [file pharmaceutics-13-00897-s001.zip › pharmaceutics-1240333.pdf]

pharmaceutics

MDPI

Supplementary Materials: Effects of Lapatinib on HER2-Positive and HER2-Negative Canine Mammary Carcinoma Cells Cultured In Vitro

Antonio Fernando Leis Filho, Patrícia de Faria Lainetti, Priscila Emiko Kobayashi, Carlos Eduardo Fonseca Alves and Renée Laufer Amorim

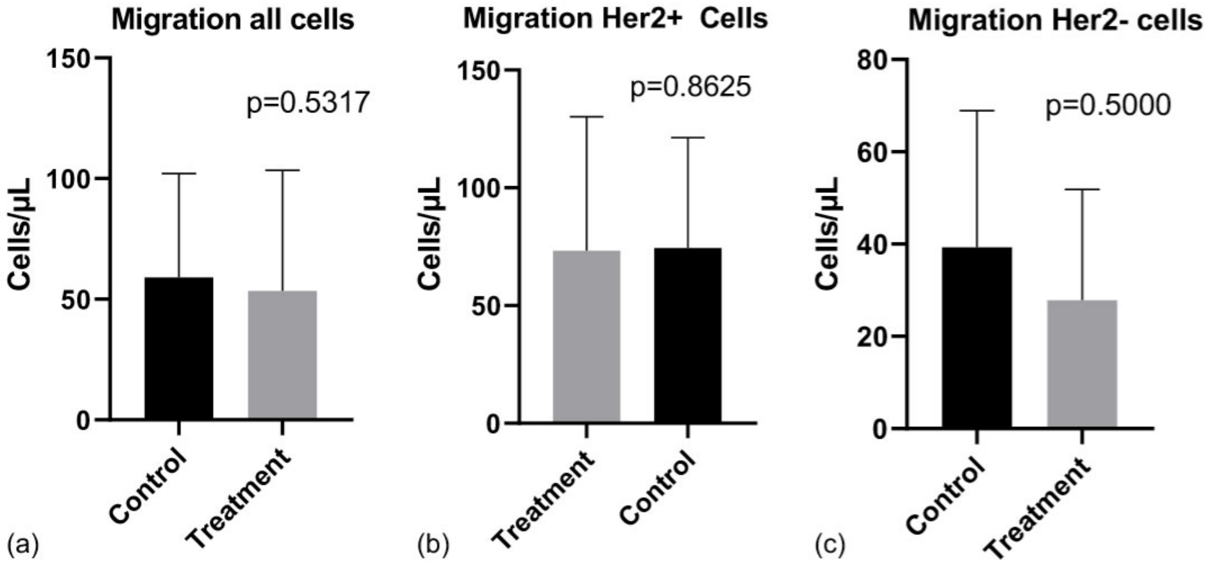

Figure S1. Mann-Whitney analysis of cell migration. There was no statistical difference in any analysis (a): All cell cultures. (b): HER2+ cell cultures. (c): HER2− cell cultures.

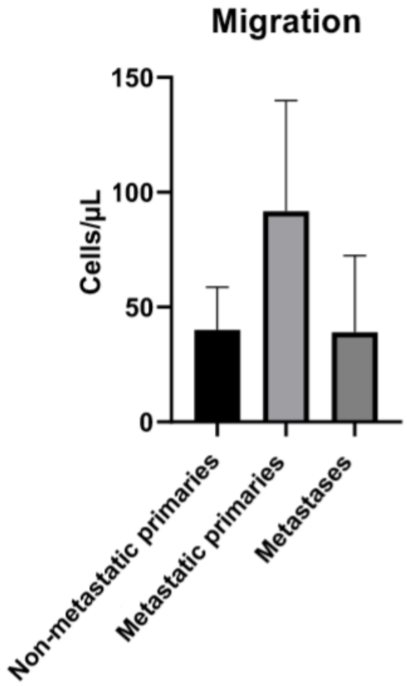

Figure S2. ANOVA evaluation of cell culture migration when grouped into non-metastatic primaries, metastatic primaries and metastases. Non-metastatic primaries versus metastatic primaries p = 0.2873; Non-metastatic primaries versus metastases p = 1; Metastatic primaries versus metastases p = 0.1822.

Table S1. Clinical data from animals with mammary carcinoma used for primary cell culture (Lainetti *et al*., 2020).

**Identification**	**Breed**	**Age (Years)**	**Histologic classification**[1]
UNESP-CM1	Poodle	12	Solid carcinoma
UNESP-CM5	Teckel	10	Solid carcinoma
UNESP-CM9	Mixed breed	12	Solid carcinoma
UNESP-CM60	Teckel	14	Adenosquamous carcinoma
UNESP-MM1	Poodle	12	UNESP-CM1 bone metastasis
UNESP-MM4	Teckel	14	UNESP-CM60 lymph node metastasis

[1] Neoplasms were classified and graded following Goldschmidt et al., 2011.
